# Exploring Pattern of Relapse in Pediatric Patients with Acute Lymphocytic Leukemia and Acute Myeloid Leukemia Undergoing Stem Cell Transplant Using Machine Learning Methods

**DOI:** 10.3390/jcm13144021

**Published:** 2024-07-10

**Authors:** David Shyr, Bing M. Zhang, Gopin Saini, Simon C. Brewer

**Affiliations:** 1 Department of Pediatrics, Division of Pediatric Hematology/Oncology, Section of Stem Cell Transplant, Stanford University, Stanford, CA 94305, USA; 2 Department of Pathology, Stanford University School of Medicine, Stanford, CA 94305, USA; 3 Stem Cell and Gene Therapy Clinical Trial Program, Department of Pediatrics, Stanford University, Stanford, CA 94305, USA; 4 Department of Geography, University of Utah, Salt Lake City, UT 84112, USA

**Keywords:** leukemia, relapse, predictive model, random forest, machine learning

## Abstract

**Background**. Leukemic relapse remains the primary cause of treatment failure and death after allogeneic hematopoietic stem cell transplant. Changes in post-transplant donor chimerism have been identified as a predictor of relapse. A better predictive model of relapse incorporating donor chimerism has the potential to improve leukemia-free survival by allowing earlier initiation of post-transplant treatment on individual patients. We explored the use of machine learning, a suite of analytical methods focusing on pattern recognition, to improve post-transplant relapse prediction. **Methods**. Using a cohort of 63 pediatric patients with acute lymphocytic leukemia (ALL) and 46 patients with acute myeloid leukemia (AML) who underwent stem cell transplant at a single institution, we built predictive models of leukemic relapse with both pre-transplant and post-transplant patient variables (specifically lineage-specific chimerism) using the random forest classifier. Local Interpretable Model-Agnostic Explanations, an interpretable machine learning tool was used to confirm our random forest classification result. **Results**. Our analysis showed that a random forest model using these hyperparameter values achieved 85% accuracy, 85% sensitivity, 89% specificity for ALL, while for AML 81% accuracy, 75% sensitivity, and 100% specificity at predicting relapses within 24 months post-HSCT in cross validation. The Local Interpretable Model-Agnostic Explanations tool was able to confirm many variables that the random forest classifier identified as important for the relapse prediction. **Conclusions**. Machine learning methods can reveal the interaction of different risk factors of post-transplant leukemic relapse and robust predictions can be obtained even with a modest clinical dataset. The random forest classifier distinguished different important predictive factors between ALL and AML in our relapse models, consistent with previous knowledge, lending increased confidence to adopting machine learning prediction to clinical management.

## 1. Introduction

Acute leukemia is the most common form of childhood cancer, accounting for 25% of all cancer before the age of 20; acute lymphoblastic leukemia represents most of the cases at around 80% while acute myeloid leukemia (AML) represents 15 to 20% of all the cases [1,2]. While most patients can be treated with chemotherapy, some require hematopoietic stem cell transplant (HSCT) for durable remission in which leukemia relapse still remains a major cause of treatment failure [3]. Post-transplant donor chimerism has been identified as a predictor of relapse in patients with hematologic malignancies who received HSCT, suggesting the possibility of meaningful relapse surveillance prior to the detection of minimal residual disease [4,5,6,7,8]. The application of these post-transplant chimerism analyses to predict leukemia relapse is limited by the fact that studies mostly correlate relapse to a single selected threshold of chimerism at fixed time points, making it more difficult to apply to individual patients and their own unique risk factors. The ability to forecast more accurately leukemia relapse would likely improve post-transplant outcome by enabling more timely initiation of post-transplant treatment strategies, including hypomethylating agents, targeted therapies like inhibitors of FLT3, Hedgehog and Menin, as well as cellular therapies like donor lymphocyte infusion and allogeneic chimeric antigen receptor T cells [9,10,11,12]. A prediction model that takes account of both pre-transplant risk factors and post-transplant chimerism could help clinicians assess relapse risk and individualize treatment strategies.

One way to achieve a deeper understanding of the complex interaction of multiple risk factors is through the application of machine learning (ML), a suite of data analysis methods which automate analytical model building emphasizing pattern recognition and which has been adopted in hematology [13,14,15,16]. Instead of focusing on making statistical inference to the entire population, ML methods hone in on the structure of the data itself, leading to better pattern recognition [17]. A number of studies have used ML to build predictive models with large datasets; Fuse et al. published their work on using ML to predict leukemia relapse within the first year of HSCT and achieved a highly accurate prediction in cross validation with only seven pretransplant variables [18]. On a larger scale, Shouval et al. reported the European Society for Blood and Marrow Transplantation (EBMT) ML analysis of Day 100 non-relapse mortality and found that only three to five variables were necessary to achieve maximum predictive skill in each model, suggesting that a few high impact variables might be adequate to make accurate predictions for focused clinical questions with ML [19]. While there are increasing numbers of large or “big” datasets, there are many more smaller datasets available from more focused clinical studies. These findings raise the question of whether ML methods, while well suited for “big data”, may also be successfully applied to smaller datasets to extract useful information and build useful predictive models that could complement standard statistical analysis [20,21,22,23]. An obstacle of applying ML in clinical decision making is that these prediction models are often viewed as a “black box” to clinicians. Interpretable machine learning (IML) methods can help illustrate in an intuitive manner on how an ML algorithm learns the relationship between the input variables and the predicted outcome [24,25]. To demonstrate further the potential of ML/IML analysis to extract valid information for focused clinical questions, we used a random forest (RF) classifier, an ML algorithm, to analyze the pattern of post-transplant relapse using pre-transplant variables and post-transplant chimerism in a single-center cohort of patients with ALL and AML undergoing HSCT. Our RF analysis yielded findings consistent with the current knowledge, but was also able to detect different patterns of interactions between the variables in subgroups. IML, specifically Local Interpretable Model-Agnostic Explanations (LIME), was applied to each individual patient and collaborated with our RF analysis, lending more confidence in the RF prediction model [26].

## 2. Materials & Methods

### 2.1. Study Design and Data Acquisition

We performed a retrospective archival data analysis using RF classification to produce predictive models of leukemic relapse in post-HSCT setting. Patients with the diagnosis of ALL and AML (confirmed by immunophenotyping and pathology review) undergoing HSCT with at least 18 months of follow-up at Lucile Packard Children’s Hospital (LPCH), a tertiary teaching hospital, from 2012 to 2020 were included in the analysis. As most leukemic relapses occur within the first 2 years post-SCT, patients who relapsed beyond 24 months post-HSCT were excluded from the analysis [27]. This was also due to the paucity of chimerism data beyond the first year of HSCT and the increased number of patients lost to follow up. Patients who had no chimerism data were also excluded. Time-invariant variables included demographics, remission status, inclusion of total body irradiation in the conditioning regimen, clinical diagnosis of the graft-versus-host (GVHD) diseases post-HSCT. Graft source, HLA match, and GVHD prophylaxis were combined as one variable following the principle of dimensionality reduction [28] given our center’s standardized approach—bone marrow grafts (mostly 10/10 HLA match, only five patients had 9/10 HLA matched donors) were given tacrolimus/methotrexate, cord blood grafts were given tacrolimus/mycophenolate, and peripheral blood stem cell grafts were almost exclusively used in haploidentical SCT with ex vivo T-cell depletion. Post-HSCT donor chimerism tests were performed between 1 and 5 times (at approximately 1, 2, 3, 6, and 12-months post-transplant) per patient and used as time-variant variables. Leukemic relapse was defined as detection of leukemic blasts by minimal residual disease flow cytometry and confirmed by the pathology review. Chimerism results at the same time of the leukemic relapse were excluded since they would have correlated 100% with relapse and offer no value in the predictive model.

Post-transplant chimerism was performed with the AmpFLSTR™ Identifiler™ PCR Amplification Kit (Thermo Fisher Scientific, Waltham, MA, USA) which is a multiplex short tandem repeat assay. The test involves amplifying 15 tetranucleotide repeat loci and amelogenin gender determining marker in a single PCR reaction using DNA extracted from peripheral blood/bone marrow aspirate samples, and lineage-specific cell subsets (CD3, CD15, and CD34) isolated from blood/marrow specimens [29]. For isolation of CD34+ cells from peripheral blood or bone marrow, Ficoll–Hypague was first used to isolate the mononuclear cells followed by positive selection of CD34+ cell subset with CD34 monoclonal antibody conjugated to magnetic nanoparticles. The rest of the cell subsets were isolated through positive selection with the corresponding monoclonal antibodies conjugated to magnetic nanoparticles. Based on the differences between recipient and donor STR alleles, the presence and quantitative fractions of recipient/donor chimerism were determined.

Clinical data were collected from an internal database maintained for the Center of International Blood Marrow Transplant Research data submission by the data management team at LPCH and cross checked against source documents in the electronic medical record (EMR). Chimerism data were obtained from EMR and cross checked with the internal HLA laboratory database for accuracy and completeness. All data collected were independently verified by two investigators.

Once the data were verified and cleaned, they were assembled and merged for machine learning. For the analysis, we considered each test to be an independent observation, with any patient-level impact on relapse probability being controlled by the time-invariant variables. Each set of test results is linked to the final relapse outcome for that patient, coded as a ‘1’ for relapse, and ‘0’ for no relapse within the study period. As we excluded any post-relapse test information, our model framework estimates the risk of future relapse, given any test within the 18-month follow-up period.

The institutional review boards of both Stanford (IRB 58403, approval date 5 December 2020) and University of Utah (IRB 00137615, approval date 26 October 2020) approved the study. This study received Stanford and Utah IRB approval for a waiver of informed consent since this was submitted as a retrospective data study.

### 2.2. Standard Statistical Analysis

Wilcoxon rank sum test was performed on all post-transplant lineage-specific donor chimerism samples, analyzing them collectively as well as at different time points. This was performed to confirm the correlation between mixed chimerism and relapse in our dataset prior to machine learning analysis.

### 2.3. Machine Learning Analysis

For the relapse dataset, we first created a simple baseline predictive model to compare against the random forest; we defined the baseline model by setting the probability of relapse ($p_{relapse}$) for each case as the proportion of relapse cases in the sample dataset. We further used a Monte Carlo approach to assess the uncertainty in this baseline model by repeatedly assigning relapse or non-relapse status to each case based on random draws from a binomial distribution with ${p=p}_{relapse}$. This Monte Carlo simulation provided a range of possible outcomes and their probability of occurring for any test.

We then used a random forest to build a predictive model of relapse risk. Model predictive skill was assessed using a nested 5-fold cross-validation. The outer cross-validation loop was used to assess model skill as the area under the curve (AUC) score of the receiver operator characteristic (ROC) curve, a threshold independent metric widely used to test the accuracy of binary predictions, as well as sensitivity and specificity. In addition to this outer cross-validation, we used an inner 5-fold cross-validation to tune the model hyperparameters, including the minimum node size (*nodesize*), the size of the subset of variables for each split (*mtry*), and the number of trees (*ntree*). A stratified sampling approach was used to form all training and testing datasets to ensure that all tests for an individual patient were either in the training or testing dataset. The same approach was used to assess the baseline model to allow comparisons with the random forest results.

Following cross-validation, we built a final random forest model using the full dataset and the parameter values obtained from tuning. This model was then used to estimate feature importance, partial dependencies that show the marginal response of the model, and LIME models. All calculations were carried out using the open-source R statistical language, which provides many add-on packages to facilitate machine learning [30]. Random forests were built using the R package *ranger*, which allowed the forest to be built using multiple CPU cores, with notable increases in computational time [31]. Cross-validation was carried out using the *caret* package [32], and LIME models were built using the *lime* package. Our work contains all pertinent elements of medical AI publication per International Journal of Medical Informatics 2021 guidelines [33].

### 2.4. Data Sharing

Please contact the corresponding author for the patient data set. Machine learning codes with explanation as well as all subgroup analysis and LIME analysis for every individual patient can be found at https://simonbrewer.github.io/aml_all/ (accessed on 6 July 2024).

## 3. Results

**Figure 1:**
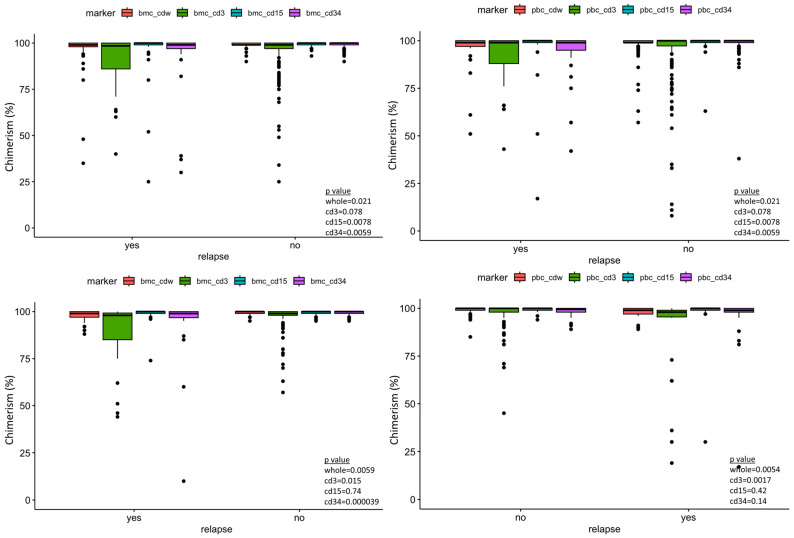
Box plot of the post-transplant lineage-specific donor chimerism showing significant difference of peripheral blood CD34 donor chimerism between relapse and no-relapse, taking all measurements together as well at the different time points post-transplant. Bmc—bone marrow chimerism, pbc—peripheral blood chimerism, cdw—whole. Top left panel—ALL bone marrow chimerism. Top right panel—ALL peripheral blood chimerism. Bottom left—AML bone marrow chimerism. Bottom right—AML peripheral blood chimerism.

Sixty-three ALL patients and 46 AML patients were included in our data analysis, with a total of 141 tests. Table 1 and Table 2 summarize the variables included in our study. The median and mean days from the last peripheral chimerism to relapse in our study cohort were 63 and 129 days for ALL, and 39 and 132 days for AML, respectively. All patients had minimal residual disease evaluation by flow cytometry on the same days of the chimerism testing. Wilcoxon rank sum test demonstrated statistical significance between peripheral blood and marrow donor chimerism between the relapse vs. no-relapse group in both the ALL and AML cohort (Figure 1).

We observed a drop-off in the post-HSCT chimerism testing for both ALL and AML cohorts starting at the 6-month time points and significantly less at the 12-month time points. Table 2 shows, for ALL, only 62% of peripheral blood and 68% of bone marrow chimerism data (defined as the number of available chimerism measurements/the number of expected chimerism measurements of the surviving cohort at specific time point) at 6 months post SCT while even less is available at 12 months (PB 37%, BM 22%). A similar pattern was observed on the AML cohort as well—PB 50%, BM 67% at the 6-month time point; PB 37%, BM 41% at the 12-month time point. This is partially attributed to deaths from other causes and relapses. The missing chimerism data are likely to have minimal impact on the model given that most of the relapses occur within 300 days (12 out of 14 ALL relapses and 10 out of 13 from AML as shown in Table 1).

Hyperparameter tuning of the random forest resulted in the following values: *mtry* (number of variables randomly chosen for each split) = 8; *nodesize* (minimum node size for partitioning) = 4; *ntree* (total number of trees built) = 500. Model accuracy was first assessed using the out-of-bag (OOB) error rate estimate, based on the samples excluded in each bootstrap iteration. The OOB rate was 8% for the ALL cohort and 14% for the AML cohort, which supported strongly the validity of our models. The results of the cross-validation process showed that a random forest model using these hyperparameter values achieves 85% accuracy, 85% sensitivity, 89% specificity for ALL and 81% accuracy, 75% sensitivity, and 100% specificity for AML at predicting relapses within 24 months post-HSCT in cross validation. This represented a significant improvement over the baseline Monte Carlo simulation model, which has sensitivity similar to the incidence of relapse in our patient cohorts.

**Figure 2:**
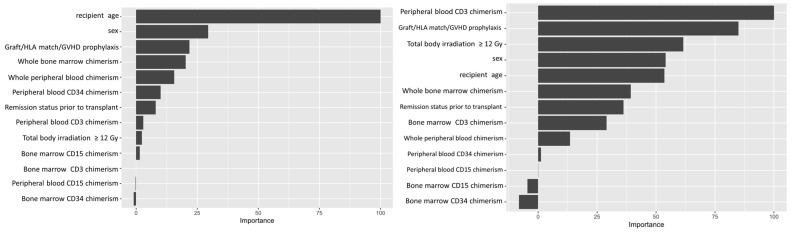
Variable importance plot. Left panel shows variable importance for ALL. Right panel shows variable importance for AML. Recipient age at time of transplant was the most important variable (or feature) in the random forest classification for leukemic relapse for ALL. Peripheral CD3 donor chimerism was the most important variable for AML.

Variable importance values were estimated using a final random forest model based on the full dataset and the selected hyperparameter values, and ranked based on the relative importance of the different variables. The importance value of different variables is conventionally normalized to the most important variable, which is expressed as 100. This allows easy visualization of relative strengths of the variables tested. For the ALL cohort, our analysis showed recipient age as the most important predictive feature amongst the variables we tested, while whole blood or marrow chimerism was the most important post-transplant variable. For AML, peripheral CD3 chimerism was the most important variable (Figure 2).

**Figure 3:**
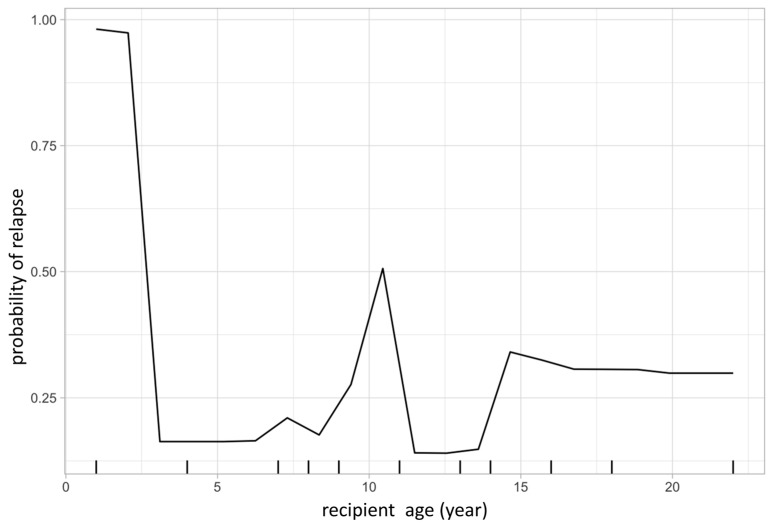
Partial dependence plot visualizes the relationship of a given variable (feature) to leukemic relapse. In this figure, X-axis represents recipient age at transplant and Y-axis represents the probability of leukemic relapse as predicted by the random forest classification. Recipient age at transplant shows a bimodal distribution with younger patients having lower risk of relapse except those less than 1-year old, representing the very high-risk infantile leukemia.

**Figure 4:**
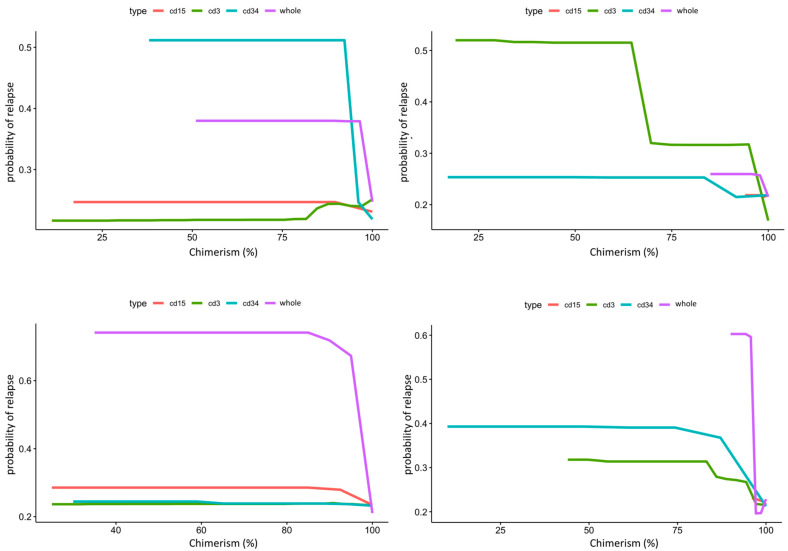
Composite partial dependence plot of CD3, CD15, CD34, and whole peripheral/marrow blood donor chimerism to leukemic relapse. In this figure, X-axis represents lineage specific donor chimerism at transplant and Y-axis represents the probability of leukemic relapse. Top panel shows peripheral blood chimerism partial dependence for ALL on the left and AML on the right. CD34 chimerism below 95% dramatically increases risk of relapse for ALL, while CD3 chimerism below 95% dramatically increases risk of relapse for AML. Bottom panel shows bone marrow chimerism partial dependence for ALL on the left and AML on the right. Whole marrow chimerism below 95% greatly increases risk of relapse for both ALL and AML.

The same model was then used to calculate partial dependency plots (PDPs) for the highest ranked variables from the importance analysis. The PDP of the probability of relapse to recipient age at transplant is shown in Figure 3, and the PDP of peripheral blood chimerism of various lineages in Figure 4. The age PDP identified the highest risk of relapse in patients less than 2-years old in our cohort (reflecting the very high-risk infant ALL patients) and higher relapse risk in teenage/adolescent patients in ALL.

PDPs for the lineage specific peripheral blood chimerism showed an increase in relapse risk with decreases of all lineages, but large differences in the scale response, indicating that CD34 was most impactful at predicting relapse, despite its relatively small proportion (0.01 to 0.1%) in the peripheral blood [29]. Notably, this exhibited a threshold effect at 95% donor chimerism where <95% confers significantly increased relapse risk. In contrast, age did not have a large impact in risk of relapse in AML. PB CD3 chimerism showed a similar threshold at 95%. Of note, whole marrow chimerism was most predictive of relapse compared to lineage specific chimerism in both ALL and AML patients with a threshold of 95% (Figure 4).

**Figure 5:**
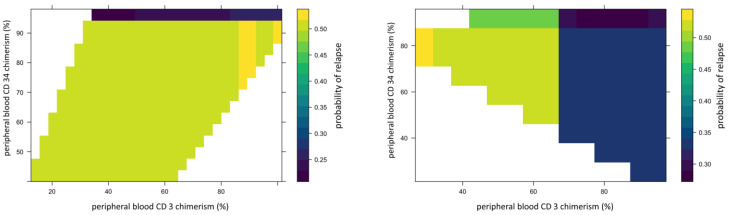
Example of 2D partial dependence plot showing interaction between peripheral blood CD34 donor and CD3 chimerism. In this figure, X-axis represents peripheral blood CD34 donor chimerism and Y-axis represents peripheral blood CD3 donor chimerism. The heat map scale represents updated probability of relapse combining the two variables. The left panel shows in ALL, >95%. Peripheral blood CD34 donor chimerism lowers the probability of relapse for recipient regardless of donor CD3 chimerism. The right panel shows stronger impact of donor CD3 chimerism than donor CD34 on risk of relapse in AML.

We used 2-dimensional PDPs to illustrate the interaction between two continuous variables. These illustrated changes in relapse risk are shown as a heatmap, with the color scale indicating the probability of relapse for pairwise combinations of two selected variables. The interaction between peripheral blood CD34 and CD3 chimerism (Figure 5) showed that the 95% threshold of CD34 chimerism values far outweighs the effect of changes in CD3 chimerism in ALL and vice versa in AML.

**Figure 6:**
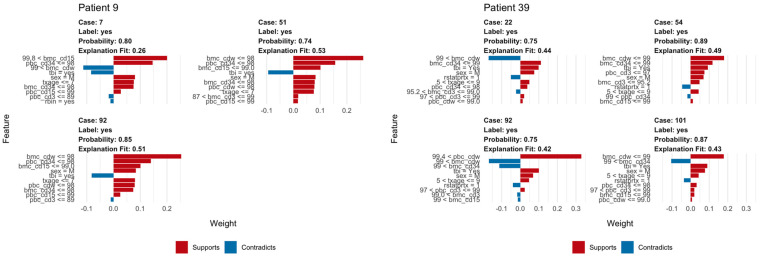
Example of LIME analysis. The left panel shows LIME analysis for a patient with ALL; note TBI in conditioning regimen consistently acts as opposing factor for relapse. The right panel shows how similar LIME reveals the logic of the random forest classifier in making a prediction of relapse in a patient with AML including whole bone marrow chimerism below 99% and peripheral blood CD3 chimerism below 93%.

We used LIME plots to illustrate how the random forest model makes predictions for an individual, helping to identify how the specific characteristics of an individual increase or decrease the probability of predicted relapse. As the random forest model captured both non-linearities in the data and interactions between variables, the impact of these characteristics may vary strongly between individuals. For example, an individual’s age might correspond to a region of the partial dependency where the response changes very little, so a change in the patient’s age would have little impact on the overall prediction. As a result, other variables were able to dominate and inform the prediction. In each plot, the bars show whether the value of a variable decreases (red) or increases (blue) the risk of relapse. In a given example, the overall predicted probability or relapse is given above each figure and increases slightly and is expressed in probability (Figure 6).

The explanation fit indicates how well the LIME analysis explains the model predictions for that individual; lower values indicate that the results should be interpreted with caution. Our LIME analysis has explanation fit up to 0.5 for both ALL and AML, indicating the LIME can explain about 50% of the RF model. The LIME analysis for consistently selected TBI = ‘yes’, having a positive effect at decreasing relapse risk for ALL patients, in contrast with the variable importance scores (Figure 2), where TBI was only ranked ninth, far behind age, the most important variable. This indicated that while the presence or absence of TBI has relatively little predictive power for the entire cohort, it may be highly important for individual patients.

## 4. Discussion

A major obstacle of applying ML in the clinical setting is that it can often be viewed by clinicians in terms of “black box” models, in which the ML algorithm make predictions in an unknown fashion, leading to skepticism. On the contrary, ML methods can actually provide information about datasets both “globally” and “locally” in an intuitive manner, and we argue that this information can be used to supplement traditional assessment methods, even with small datasets. While many ML algorithms can be used to make a predictive model, we selected the random forest classifier to build our predictive model since it is based on decision tree analysis, which is easy to understand intuitively. Variable importance values were estimated using a final random forest model based on the full dataset and the selected hyperparameter values, and ranked based on the relative importance of the different variables. Partial dependence allows us to visualize how the probability of relapse varies across the range of values for any variable, illustrating how the ML can capture different types of response, including linear, non-linear, or threshold. By calculating the partial dependency for two variables, we can further visualize how the model captures interactions between variables and that these interactions can also be non-linear. It is important to note that while we restricted our results to first-order interactions, the model includes higher order interactions (e.g., the influence of variable *x*_3_ on the interaction between *x*_1_ and *x*_2_) [34]. These plots showed the nuance of the different variables and their interactions to relapse risk, which was the basis of improved prediction.

In contrast to the “global” view provided by variable importance and partial dependence, IML, e.g., LIME, shows how predictions are made “locally” or for individual cases. This provides another check on the logic of the machine learning prediction, and helps to identify the factors that are most relevant to an individual prediction, and the extent to which they increase or decrease the relapse risk as well as to how confident we can be with any individual prediction. While the LIME analysis did not create the same variable importance as RF, it identified many of the same important variables as RF, which increased our confidence in the RF model. This again highlights the nonlinearity and interactions captured by the model, and allows us to appreciate better the main drivers of a given case which may be different between cases, and notably, different from what is the most important variable for the population. Our analysis suggests PB CD34 donor chimerism might warrant further investigation for pediatric patients with ALL for relapse surveillance. PB CD3 chimerism might be valuable in AML patients post-HSCT; however, the brisker pace of AML relapse makes disease surveillance challenging, regardless.

The RF classifier dramatically improved relapse prediction within 24 months post-HSCT in the context of our dataset. Our model is far from being perfect and as it is based on a small sample size and a modest number of variables, it is difficult to know how well this would generalize to a patient population outside of our institution. However, the goal of our study was not meant to create a definitive or generally applicable prediction model, but instead to demonstrate the rationale of adopting ML/IML more broadly to improve patient care and add more value to existing data and knowledge, even within a single institution. It is worth noting that during our study, the analytical results changed as we included more patient data, further illustrating the adaptable, learning nature of ML. ML methods offer a highly flexible approach for working with complex datasets, including low *n*, high *p* data, in which the number of variables or *features* is greater than the number of observations. The addition of more objective measures including biomarkers and biopsy finding for GVHD and immune reconstitution could further improve our model as these events have strong biological relevance to leukemic relapse. Similarly, pharmacokinetic data of conditioning chemotherapy (ATG, busulfan) and pre-transplant next generation sequencing minimal residual disease testing have recently been shown to play a bigger role in relapse and can also be incorporated into our existing model [35,36]. Furthermore, inclusion of pre-transplant chemotherapy treatment data, specifically salvaged treatment used to achieve remission prior to SCT, will likely further improve our model and shed more light on the optimal salvage strategy. ML analysis is potentially a useful and complementary approach to standard statistical methods on clinical studies constrained by smaller patient sample size or under powered [37]. The common perception of “large” sample size is requisite for validity or applicability in ML and should be re-evaluated. The dataset should be appropriate for the clinical challenge and might not necessarily need to be large or with highly granular details but, more importantly, relevant. Machine learning is not only for “big data”, but can also be applied to smaller datasets, which may be more appropriate to answer more focused questions, particularly with rare diseases and less common medical challenges.

## Figures and Tables

**Table 1:** Patient and transplantation characteristics.

	ALL (n = 63)	AML (n = 46)
Age (year)		
Range	1 to 22	1 to 23
Median	12	11
Mean	11	10
Sex (n)		
Male	37 (59%)	29 (63%)
Female	26 (41%)	17 (27%)
Pre-transplant Remission Status (n)		
Complete Remission 1	21 (33%)	32 (70%)
Complete Remission 2	36 (57%)	10 (22%)
Complete Remission 3	6 (10%)	0
Relapse	0	3 (7%)
Unknown	0	1 (2%)
Graft Source (n)		
Bone Marrow	37 (59%)	22 (48%)
Cord Blood	8 (13%)	9 (20%)
Peripheral Blood Stem Cell	18 (28%)	15 (32%)
Total Body Irradiation (n)		
Yes	57 (90%)	25 (54%)
No	6 (10%)	21 (46%)
Acute Graft versus Host Disease any grade (n)		
Yes	38 (60%)	22 (48%)
No	25 (40%)	24 (52%)
Relapsed		
Yes (n)	14 (26%)	13 (28%)
Range (days)	53 to 620	54 to 621
Mean (days)	244	210
Median (days)	188	174
Days from last peripheral blood chimerism		
Range (days)	7 to 531	15 to 467
Mean (days)	129	132
Median (days)	63	39

ALL relapses days post SCT—55, 56, 59, 110, 145, 146, 173, 203, 219, 230, 377, 448, 584, 620. AML relapses days post SCT—54, 62, 77, 91, 126, 159, 174, 187, 211, 228, 362, 389, 621.

**Table 2:** Chimerism data, TX-transplant.

	Peripheral Blood			Bone Marrow		
ALL	Number of Tests (n, % Data Present)	Mean (Post-TX Days)	Range (Post-TX Days)	Number of Tests (n, % Data Present)	Mean (Post-TX Days)	Range (Post-TX Days)
Chimerism #1	55 (87%)	27	12 to 40	63 (100%)	21	24 to 62
Chimerism #2	49 (78%)	60	30 to 91	59 (94%)	63	43 to 98
Chimerism #3	47 (74%)	100	42 to 186	55 (87%)	96	77 to 186
Chimerism #4	39 (62%)	191	84 to 384	43 (68%)	180	127 to 377
Chimerism #5	23 (37%)	332	139 to 532	14 (22%)	321	173 to 449
AML						
Chimerism #1	36 (78%)	28	12 to 37	42 (91%)	32	21 to 43
Chimerism #2	28 (61%)	61	29 to 85	44 (95%)	63	43 to 89
Chimerism #3	26 (57%)	97	62 to 145	36 (78%)	93	69 to 119
Chimerism #4	23 (50%)	174	97 to 265	31 (67%)	174	119 to 197
Chimerism #5	17 (37%)	345	243 to 518	19 (41%)	335	182 to 557

## Data Availability

Please contact the corresponding author for the patient data set. Machine learning codes with explanation as well as all subgroup analysis and LIME analysis for every individual patient can be found at https://simonbrewer.github.io/aml_all/ (accessed on 6 July 2024).
